# Managerial features and outcome in neonatal intensive care units: results from a cluster analysis

**DOI:** 10.1186/s12913-020-05796-0

**Published:** 2020-10-16

**Authors:** Simone Fanelli, Roberto Bellù, Antonello Zangrandi, Luigi Gagliardi, Rinaldo Zanini

**Affiliations:** 1 Department of Economics and Management, University of Parma, Via J. F. Kennedy, 6, Parma (PR), Italy; 2 Neonatal Intensive Care Unit, ASST of Lecco, Lecco, Italy; 3 Division of Pediatrics and Neonatology, Versilia Hospital, AUSL Toscana Nord Ovest, Viareggio, Italy

**Keywords:** Healthcare management, Healthcare organisation, Managerial model, Cluster analysis, Neonatal outcome, NICU, Italy

## Abstract

**Background:**

Healthcare organisations differ in performance even if they are located in the same country or region. Suitable managerial practices and organisational processes can lead to better health outcomes. As a result, hospitals are constantly looking for managerial arrangements that can improve outcomes and keep costs down. This study aims to identify different managerial models in neonatal intensive care units (NICUs) and their impact on a large number of outcomes.

**Methods:**

The research was conducted in Italy, within the SONAR project. SONAR’s aim was to identify the characteristics of NICUs, monitor outcomes and promote best practices. This study includes 51 of the 63 NICUs that took part in the SONAR project. Questionnaires on the activities and managerial features were administered to doctors and nurses working in NICUs. A total of 643 questionnaires were analysed from doctors and a total of 1601 from nurses. A cluster analysis was performed to identify managerial models of NICUs.

**Results:**

Three managerial models emerged from cluster analysis: traditional, collaborative and individualistic. In the “traditional” model the doctor is above the nurse in the hierarchy, and the nurse therefore has exclusively operational autonomy. The “collaborative” model has as key elements professional specialisation and functional coordination. The “individualistic” model considers only individual professional skills and does not concern the organisational conditions necessary to generate organisational effectiveness.

The results also showed that there is an association between managerial model and neonatal outcomes. The collaborative model shows best results in almost all outcomes considered, and the traditional model has the worst. The individualistic model is in the middle, although its values are very close to those of traditional model.

**Conclusions:**

Health management needs to assess NICU strategically in order to develop models to improve outcomes. This study provides insights for management useful for designing managerial characteristics of NICUs in order to achieve better results. NICUs characterised by a collaborative model in fact show better neonatal outcomes.

## Background

Healthcare organisations differ in performance even if they are located in the same country or region [1]. Suitable managerial practices and organisational processes can lead to better health outcomes and, conversely, managerial and organisational disorder may be related to lower quality of care [2, 3].

As a result, hospitals are constantly looking for managerial arrangements that can improve outcomes and keep costs down. In hospitals, the management of neonatal intensive care units (NICUs) is particularly complex and expensive [4]. NICUs in fact deal with infants who are very vulnerable due to their age and conditions and who require intensive care. Within NICUs, preterm newborns particularly are recipients of intensive care, together with term newborns affected by congenital malformation or birth asphyxia. According to the World Health Organisation (WHO), preterm birth is a worldwide challenge: 11.1% babies annually are born preterm worldwide (about 15 million babies); prematurity is the first cause of death for neonates; many preterm babies who survive face a lifetime of disability; and countries with health systems with low human and financial resources have higher mortality rates [5–7]. As for costs, Zupancic has estimated costs ranging from $76,000 to $159,000 per preterm baby in NICU [4].

Identifying managerial models which improve NICU performance is an important challenge for hospitals around the world. Literature on quality improvement in health is unanimous that several managerial conditions are necessary to generate better performance, and this is true at the level of hospital and single unit level [8–10]. Nevertheless, most of the studies in literature have focused on one single managerial feature and its impact on specific outcomes in NICUs. For example, Rogowski et al. investigated the relationship between nurse staffing and infection rate [11]; Miedaner et al. explored the influence of employee’s commitment on quality of care [12]; Pollack and Kock studied the association between leadership and outcome [13]; and Welp and Manser examined clinician occupational well-being and patient safety [14].

There is therefore a need to identify how different managerial features of NICUs impact on a wider spectrum of neonatal outcomes. To overcome this research gap, this study aims to identify different managerial models in NICUs and their impact on a large number of outcomes. In this study we hypothesise that managerial models were associated with neonatal outcomes.

The findings of the research should be useful to health management in designing NICU managerial features in order to achieve better results in terms of outcome.

### The assessment of the managerial model

Several assessment systems of managerial variables exist in the literature. This study uses the system developed by Øvretveit for WHO [8]. Many scholars consider the framework described in the WHO study as a positive example for defining managerial features to improve health outcomes [15–17], although it requires adaptation to specific contexts [18–20].

The WHO study by Øvretveit finds that five areas must be assessed by managers to ensure quality care in a hospital [8]. These areas are briefly described below.

#### Area 1. Performance and results review

The improvement of quality performance in hospital is based on systematic reviews of performance and results. Indicators and measures should be used to evaluate activity volumes, the complexity of case studies, and the level of absorption of resources, etc. Management should develop and implement appropriate tools to detect these elements [21, 22].

#### Area 2. Benchmarking

Benchmarking is the practice of comparing performance metrics to hospital bests and best practices from other medical units. Dimensions typically measured are quality of care and costs. Managers are responsible for carrying out benchmarking in order to trigger continuous improvement processes [23, 24].

#### Area 3. Leadership

Stefl defines leadership as “the ability to inspire individual and organisational excellence, to create and attain a shared vision, and to successfully manage change to attain the organisation’s strategic ends and successful performance” [25]. It is one of the most important skills required by managers. Leadership is essential in creating favourable conditions and leading employees toward the desired results [22].

#### Area 4. Clinical guidelines, protocols and procedures

Health managers should use guidelines, protocols and procedures to reduce variability and increase coordination between behaviours. These tools are useful both for healthcare professionals and managers. Professionals refer to protocols when basing clinical activities on scientific evidence. Managers define organisational procedures in order to coordinate actions, collect data and prevent errors [26, 27].

#### Area 5. Staff satisfaction

Many studies have shown that employee satisfaction is a key element of performance [28, 29]. Consequently, managers should devote part of their efforts to ensuring staff satisfaction. This is particularly critical in NICUs because the type of cases treated generates high levels of stress for healthcare professionals. Managers should therefore monitor satisfaction levels within the operating unit in order to reduce phenomena such as operator burnout [30].

For each of these five areas managers can make different choices, and thus design management models which are very different from each other.

This study identifies a managerial variable within the NICU for evaluation for each of the five areas. The evaluations of these variables are then used to define different managerial models of the NICUs. Finally, we investigate possible associations between managerial models and neonatal outcomes.

## Methods

### Study setting

The research was conducted in Italy, within the 5-year SONAR project (Observational Study in Neonatology: Assistance and CaRe) set up in 1999 by the Italian Neonatal Network (INN). INN includes 93 NICUs with the goals of improving safety and quality of care in neonatal care by using research, training and quality improvement projects. Since 2004, INN has been part of the Vermont-Oxford Network, the most important neonatal network in the world, which includes more than 1300 hospitals across 30 countries working to continuously improve neonatal care. 63 NICUs of the INN participated voluntarily in the SONAR project. SONAR’s aim was to identify the characteristics of NICUs, monitor outcomes and promote best practices [31].

### Data collection

Our study includes 51 of the 63 NICUs that took part in the SONAR project. Operational units (OUs) that did not present complete data in managerial variables or outcomes were excluded from our sample. SONAR covered all healthcare professionals (doctors and nurses) who work in NICUs, preterm babies, and their parents. Questionnaires on the activities and managerial features of the OU were administered in written form to nurses, doctors and parents. The questionnaires differed for the three categories of respondent.

The questionnaires were developed by the Steering Committee of SONAR on the basis of a review of international literature and a meeting with health professionals. They were validated by the Advisory Board and the Steering Committee of SONAR, and pilot testing was conducted in several OU that took part in the project.

For this study we used only responses from medical and nursing staff.

The questionnaires were only administered to medical and nursing staff who had been working in the NICU for at least 3 months (see Additional files 1 and 2). Each NICU had a Local SONAR manager, belonging to the medical staff of the unit, who provided data about the activities and outcomes of its OU and was responsible for coordinating the project at local level.

### Ethical approval

The study complies with the Italian regulations on observational clinical studies (Ministerial Circular n. 6 of 2/9/2002). The Ethics Committees of each centre involved in the SONAR project approved this study.

All the respondents to the questionnaire received an information sheet, containing a clear and comprehensive definition of the characteristics and objectives of the study. All the respondents gave written consent to the processing of personal data.

### Managerial features

For each of the areas of the framework developed by Øvretveit for WHO [8], one variable was identified to describe the managerial features (MF) of the NICU. These features can be used to describe different managerial models.

#### MF1. Complexity of case

Local SONAR managers identified the acuity score for each neonate hospitalised in their own NICU. The acuity score is a tool developed by the Vermont-Oxford Network to define the type of patients admitted to the NICU, and includes five categories of increasing severity and care commitment [31, 32]. The lowest level of complexity indicates an infant who requires minimal care, while level 5 indicates an unstable infant who requires complex intensive care. Each NICU was thus assigned an acuity score given by the average of the acuity scores of all infants treated within it. NICUs that show a higher acuity score, nearer to 5, handle more complex cases on average and have a greater absorption of resources than NICUs with a lower acuity score nearer to 1.

In this study, we used the acuity score as an indicator available to managers (the head physician of the OU) for the evaluation of the complexity of NICU activities (MF1). It thus captures the first area of the managerial model: “Performance and results review”. Previous studies have used case complexity as a performance indicator [20, 33]. The United Kingdom Neonatal Staffing Study Group also suggests that case complexity, together with random chance and care quality, is one of the main elements leading to variability in outcomes in neonatal care [34].

#### MF2. Performance measurement

Doctors in their questionnaires were asked to indicate the system used in their NICU to evaluate qualitative performance by choosing one of the five following statements: 1) Quality measurement is sporadic; 2) The individual evaluates his or her own performance using information made available by the hospital; 3) Regular meetings between colleagues to discuss some clinical cases; 4) We jointly evaluate all cases not following defined procedures; 5) Periodical evaluation on quality indicators by external entities. The first and fifth statements express two extreme methods of evaluating performance. The first embodies a managerial approach poorly oriented towards quality assessment, which is left to the will of individual professionals. The fifth statement, on the other hand, presents a clear orientation of management to robust measurement systems. For each NICU, the score for the performance measurement system was defined through the evaluation expressed by its doctors. NICUs that show values nearer to 5 use more robust measurement systems than those with a score nearer to 1.

The use of performance measurement systems is the basis for activating benchmarking processes [35]. Managers can compare data collected from their measurement system with data from other hospitals, or with their own previously collected data, to develop adequate improvement processes. This study uses the type of performance measurement system (MF2) adopted to evaluate the “Benchmarking” area in the NICU management model.

#### MF3. Participation and support

The Practice Environment Scale of the Nursing Work Index (PES-NWI) is included in the questionnaire for nursing staff. PES-NWI is a tool developed by Lake for measuring the working environment and has a high degree of reliability and validity [36]. PES-NWI is made up of 31 items evaluated on a 4-point Likert-type scale, from 1 (maximum disagreement) to 4 (complete agreement). Items are grouped by Lake into five areas, two of which were used for this study. The “Nurse participation in hospital affairs” and “Nurse manager ability, leadership, and support of nurses” areas measure whether or not the hospital uses participatory leadership in managing activities. For each NICU, the average score was calculated in the two areas of the PES-NWI. An average close to 4 shows that an NICU is oriented towards participation, support and involvement of nurses. This element is used to identify the style of “Leadership” (MF3) in the NICU managerial model.

#### F4. Foundations for quality of care in nursing

Nurses are the professionals most involved in neonatal intensive care and are characterised by a very high level of skills. In PES-NWI, two items are useful for measuring NICU propensity to involve nurses more actively in care processes: 1) Use of nursing diagnoses; 2) Nursing care is based on a nursing, rather than a medical, model. These two items of the PES-NWI show to what extent the quality of care in the NICU is based on guidelines and procedures developed by nurses (F4). Together these two items can be used to measure the “clinical guidelines, protocols and procedure” area of the managerial model [37, 38]. As for the previous variable, as the value of the variable increases towards 4, the NICU propensity to use guidelines, protocols and procedures increases.

#### MF5. Motivation of medical and nursing staff

Doctors were asked to evaluate the level of satisfaction within their NICU on a four-point Likert-type scale (1: very low, 4: high), and the average level was calculated for each NICU. Motivation levels nearer to 4 reveal management ability to create a favourable working climate within the OU [39]. Managerial practices that promote job satisfaction and emotional support are also important for preventing burnout [40]. The motivation of medical and staff nursing (MF5) is thus closely linked to the last area of the managerial model: staff satisfaction.

### Neonatal outcomes

Neonatal outcomes used for this study were defined as for the Vermont-Oxford Network. Below is a brief description of each of the nine outcomes.

Mortality refers to intra-hospital mortality. Nosocomial infection indicates whether the infant has late bacterial infection (including Coagulase Negative Staphilococcus) after Day three of life. Severe Intraventricular Haemorrhage (severe IVH) indicates whether the infant has a Grade 3 or 4 periventricular-intraventricular haemorrhage. Cystic periventricular leukomalacia (PVL) refers to multiple small periventricular cysts identified on a cranial ultrasound, computerised tomography scan, or magnetic resonance imaging scan. Severe Retinopathy of Prematurity (ROP) indicates whether the infant has a stage 3, 4 or 5 ROP. Necrotizing Enterocolitis (NEC) was diagnosed at surgery, at post mortem examination, or clinically (bilious gastric aspirate or emesis, abdominal distension, occult or gross blood in stool) and radiographically (pneumatosis intestinalis, hepato-biliary gas, pneumoperitoneum). Pulmonary Bronchodysplasia (BPD) refers to oxygen at 36 weeks postmestrual age. Newborns discharged before 36 weeks do not have BPD. Morbidity indicates whether the infant survived with one of the following key morbidities: severe IVH, BPD, NEC, pneumothorax, nosocomial infection, or PVL. Human milk indicates whether the infant was discharged receiving human milk (including human milk fortifier and/or formula milk).

Outcome data were adjusted for confounders with covariance adjustment using linear regression (analysis of covariance, ANCOVA) [41], including, as for the Vermont-Oxford risk adjustment model, gestational age, gestational age squared, 1-min Apgar score, small size for gestational age (lowest 10th percentile), multiple gestation, outborn status, gender, cesarean section delivery, and presence of a congenital anomaly [42]. For each outcome the adjusted mean was calculated for each statistical unit i.e. for each NICU, and the adjusted means were used for comparison between groups.

Data on outcomes were collected by the Local SONAR manager for each NICU. Low values associated with outcomes indicate better outcomes, except for the human milk variable, where higher values are preferable.

### Data analysis

We conducted a cluster analysis to identify managerial models of NICUs. All variables MF1 to MF5 were included in the analysis to define the groups. Because outliers could significantly influence the results of the cluster analysis, variables were first standardised [43]. Patterns of respondents’ answers were then examined by applying the agglomerative hierarchical cluster analysis using the Ward method. The hierarchical cluster analysis with its dendrogram and statistics measuring the cluster fit (pseudo F-statistic) are useful to identify the number of the clusters. In a cluster analysis, in fact, the number of clusters is subjective. In our case, dendrogram and pseudo F-statistic suggested three clusters as the best option. Final clusters were thus formed using k-centered clustering with three groups. The method of Euclidean-distance was used in order to calculate differences between the variables. ANOVA was used to test whether the clusters significantly differ from each other in terms of the five managerial features used in the cluster analysis.

After forming the three managerial models, we conducted post-hoc tests (Bonferrroni) to assess differences between clusters on the neonatal outcomes.

Finally, in order to better describe the managerial models of NICUs, we also examined the association between other key elements of the NICU and cluster membership, which are useful for measuring the resources available to the NICU. The structural aspects were: size (number of beds), and human resource endowment (number of doctors and nurses per bed and per 1000 day of length of stay). To investigate differences between clusters on structural aspects we used one-way ANOVA and Bonferroni’s test.

All statistical analyses were carried out using SPSS Statistics Version 25 and were conducted using a 5% significance level.

### The NICUs of the sample

The 51 NICUs of our sample account for 81% of OUs belonging to INN, or 43% of all Italian NICUs. In Italy, NICUs can vary greatly in size and are sometimes very small. However, in terms of the number of preterm babies managed, the OUs belonging to INN deal with about 90% of all cases nationally, and the OUs in the SONAR project about 72%.

Twety-nine of NICUs in our sample are located in the north of Italy (56.9%), 6 in the centre (11.8%) and 16 in the south (31.4%). NICUs in the north account for 59.2% of the total population of existing NICUs in the north, those in the center account for 30.0% and those in the south account for 37.2%.

Overall, a total of 14,012 cases were treated in the sample NICUs during a period of 12 months (from March 2010 to February 2011), with an average of 274.75 per NICU (median: 245; Standard Deviation (SD): 145). Our study includes all infants who met the criteria of the Vermont Oxford Network Release 13.0 NOVEMBER, 2008, i.e. with a birth weight between 401 and 1500 g or with a gestational age between 22 + 0 and 29 + 6 weeks. As for number of beds, NICUs have an average of 17.18 (median: 16; SD: 8.90) and range from a minimum of 4 to a maximum of 50 (SD: 8.90). Finally, there are on average 2.92 doctors and nurses per bed (SD: 0.65), and more precisely 0.76 doctors per bed (SD: 0.43) and 1.78 nurses per bed (SD: 1.08).

In sum, these data show that Italian NICUs work with a very different sets of resources and in widely differing organisational contexts.

For the 51 NICUs in the sample, 643 questionnaires were analysed from doctors and a total of 1601 from nurses. The response rate of doctors was 96.8% and that of nurses was 96.6%.

## Results

### Managerial features in the NICUs

Table 1 shows the main descriptive statistics for the five managerial variables used in the analysis.

**Table 1:** Managerial features: descriptive statistics

	MF1	MF2	MF3	MF4	MF5
Mean	1.8514	2.5462	2.3149	2.4157	3.0788
Median	1.8446	2.6300	2.3246	2.4047	3.1000
SD	0.2100	0.6075	0.2589	0.2582	0.3905
Minimum	1.3979	1.2500	1.4829	1.9117	2.0000
Maximum	2.3333	4.0000	2.7625	3.1486	3.7300

#### MF1

On average, the cases treated in the NICUs have a medium-low level of complexity (average acuity score: 1.85): they are mainly neonates who require intermediate care and stable infants with a defined schedule with no need for significant support. Furthermore, the minimum and maximum values of the SD show no great variability between NICUs in terms of complexity of the cases treated.

#### MF2

Performance measurement systems appear to be widely underdeveloped. Both median and average values are on average lower than 3, and only 13.72% of NICUs report a score higher than 3. The figures also show that NICUs adopt very different performance measurement systems (see SD, minimum, and maximum).

#### MF3

As for the leadership, only 27.45% of the NICUs show a participatory leadership style (F3 > 2.5). A score of 2.5 is the midpoint between agreement and disagreement, and a score of 3 or greater indicates agreement that the element is present in the OU [36]. However, most NICUs are positioned below the average level (2.5), showing that leadership is poorly oriented towards the participation, support and involvement of nurses.

#### MF4

Foundations for quality of care in nursing show a more positive result than MF3, although the mean and median are still both below the average level (2.5) and only one NICU has a score higher than 3.

#### MF5

Staff are on average satisfied (3.07), although this figure cannot be considered as completely satisfactory. In fact, the 43.14% of NICUs displays a level of staff satisfaction that lies between 2 (low) and 3 (medium).

These results give rise to two preliminary considerations. The first is that NICUs show great differences in managerial features, and the second is that there are many areas for improvement, especially in variables MF2, MF3, and MF4.

### Managerial models

The three groups that emerged from the cluster analysis contain an almost equivalent number of OUs: Cluster 1 consists of 14 NICUs, Cluster 2, 18 NICUs and Cluster 3, 19 NICUs. Table 2 presents the characteristics of the three clusters based on the managerial features used in the cluster analysis. The ANOVA analysis (Table 2) shows that the clusters differ significantly in terms of all variables used in the cluster analysis, except for complexity of case (MF1). We therefore use variables MF2 to MF5 to describe groups and interpret the NICUs.

**Table 2:** ANOVA test of the managerial features included in the cluster analysis

	Cluster 1 Mean (SD)	Cluster 2 Mean (SD)	Cluster 3 Mean (SD)	ANOVA F (p-value)
MF1	1.8593 (0.209)	1.8676 (0.218)	1.8301 (0.211)	0.155 (0.857)
MF2	2.6485 (0.563)	2.9927 (0.350)	2.0478 (0.458)	20.293 (0.000)
MF3	2.0140 (0.190)	2.5462 (0.142)	2.3175 (0.129)	47.723 (0.000)
MF4	2.1603 (0.155)	2.5766 (0.244)	2.4514 (0.182)	17.451 (0.000)
MF5	3.1664 (0.254)	3.3983 (0.225)	2.7115 (0.280)	34.664 (0.000)

#### Cluster 1

The first managerial model is mainly characterised by a low level of autonomy of nurses. Both MF3 and MF4 in fact record the lowest values of all groups. In this model, which could be defined as “traditional”, the doctor is hierarchically above the nurse, who therefore has autonomy which is exclusively operational (top-down leadership approach) [44, 45]. In the traditional model, there is no need to codify nursing activities, and the protocols and procedures are all directly in the hands of the doctor, who assigns specific tasks to his/her collaborators. When this traditional managerial model prevails, nurses participate in both the OU and hospital activities to a small extent, and it is up to doctors to lead and control the treatment processes [46].

#### Cluster 2

The second managerial model shows the highest average values for all the variables used in the analysis (MF2 – MF5). Note that these variables cover all managerial aspects of a NICU. The Cluster 2 NICUs are characterised by robust performance measurement systems, by a participatory leadership style, by nursing processes in which nurses have autonomy and collaborate closely with doctors, and by high motivation levels among staff. It therefore appears that the management of these NICUs play a crucial role in overseeing all the variables that ensure quality of care, and use a global managerial approach where each healthcare professional has their own autonomy [44]. This model can be defined as “collaborative”, and its key elements are professional specialisation, professional autonomy, and functional coordination [47, 48].

#### Cluster 3

In the third managerial model, two aspects emerge: poor orientation towards performance measurement (MF2) and low levels of motivation of the medical and nursing staff (MF5). In these NICUs there is no structured assessment system, and evaluation is often left to the sensitivity of individuals. Similarly, little attention is paid to staff motivation. These characteristics are associated with a mechanistic managerial model, based exclusively on the professional skills of each profile, with no attention to the organisation and relations between the people who work in the OU. This “individualistic” model considers only individual professional skills and is not interested in the organisational conditions necessary to generate organisational effectiveness [49].

### Association of outcome with managerial models

As shown in Table 3, there is a statistically significant difference between the mortality rate in Cluster 2 and that in Cluster 1 (*p*-value: 0.007), and also a statistically significant difference between PVL outcome in Cluster 2 and in Cluster 3 (p-value: 0.019).

**Table 3:** Neonatal outcomes adjusted for severity of illness by cluster

	CLUSTER 1		CLUSTER 2		CLUSTER 3	
	Mean (SD)	95% Confidence interval (Lower - Upper)	Mean (SD)	95% Confidence interval (Lower - Upper)	Mean (SD)	95% Confidence interval (Lower - Upper)
Mortality	14.97* (2.66)	13.43–16.51	12.01* (4.52)	9.76–14.26	14.85 (4.86)	12.50–17.19
Nosocomial infection	14.76 (1.80)	13.72–15.80	13.81 (3.52)	12.06–15.57	14.41 (3.68)	12.64–16.19
Severe IVH	8.97 (1.93)	7.85–10.08	7.41 (3.11)	5.86–8.95	8.89 (3.89)	7.01–10.77
Severe ROP	8.10 (1.69)	7.13–9.08	7.03 (3.52)	5.28–8.78	8.56 (4.40)	6.44–10.68
Morbidity	39.02 (4.79)	36.25–41.78	36.76 (8.64)	32.47–41.06	38.93 (8.64)	34.77–43.09
NEC	4.12 (0.78)	3.66–4.57	4.25 (0.82)	3.84–4.65	3.93 (1.04)	3.43–4.43
PVL	5.17 (1.74)	4.17–6.18	4.79* (1.21)	4.19–5.39	6.70* (2.55)	5.47–7.92
BPD	20.81 (3.07)	19.03–22.58	19.39 (6.09)	16.36–22.42	19.01 (7.16)	15.56–22.46
Human milk	62.39 (1.57)	61.49–63.30	63.36 (2.94)	61.90–64.83	61.64 (3.62)	59.90–63.39

* Bonferroni’s test: *p*-value < 5%

In general, Cluster 2 shows best results in almost all outcomes considered (except for NEC and BPD) and Cluster 1 has the worst outcomes. Cluster 3 is in the middle, although its values are very close to those of Cluster 1.

### Association of structural aspects with managerial models

Table 4 reports the characteristics of clusters in terms of beds, doctors and nurses per bed, and doctors and nurses per 1000 days in hospital (length of stay).

**Table 4:** Structural aspects by cluster

	CLUSTER 1		CLUSTER 2		CLUSTER 3	
	Mean (SD)	95% Confidence interval (Lower - Upper)	Mean (SD)	95% Confidence interval (Lower - Upper)	Mean (SD)	95% Confidence interval (Lower - Upper)
Beds	16.214 (7.073)	12.130–20.298	17.556 (11.587)	11.793–23.318	17.526 (7.501)	13.911–21.142
Doctors per bed	0.610 (0.248)	0.460–0.760	0.891 (0.521)	0.623–1.159	0.754 (0.429)	0.547–0.961
Nurses per bed	1.871 (0.788)	1.417–2.326	1.886 (1.447)	1.142–2.631	1.610 (0.897)	1.164–2.056
Doctors per length of stay	1.788 (0.741)	1.360–2.216	2.572 (1.181)	1.985–3.159	2.161 (1.049)	1.655–2.667
Nurses per length of stay	4.909 (1.799)	3.870–5.948	4.457 (3.293)	2.819–6.095	3.569 (2.336)	2.443–4.694

Post-hoc tests display no differences between clusters on the structural aspects. However, Cluster 2 shows a supply of resources higher than Cluster 1 and Cluster 3, except for nurses per 1000 days of stay.

## Discussion

The literature agrees that appropriate managerial models can generate better outcomes [2, 3, 13, 50]. Starting from this premise, we used the framework developed by Øvretveit for WHO to identify different managerial models within Italian NICUs. Three models emerged: traditional, collaborative and individualistic. The results showed that there is an association between managerial models and outcomes. At the same time, some variables, such as staffing levels, the size of the OU and the complexity of the cases are not decisive in defining the managerial models.

Evidence that the cluster of NICUs characterised by a “collaborative” managerial model can achieve better clinical outcomes has important implications for management. These findings can in fact be useful at the multiple decision levels present in health systems in promoting specific actions to pursue the quality of care and patient safety. To achieve these objectives, the health system actors should focus their efforts on creating conditions which foster collaboration. Unfortunately, the attention of management towards improving outcomes is often focused more on structural variables, such as bed and staff [51, 52], and specific professional tasks (task orientation) [53, 54], rather than creating effective organisational conditions (orientation organisational and managerial practices).

Below are the main implications of our study for three categories of management.

The first category is top management, responsible for the entire hospital and charged with setting general organisational policies for all OUs, including NICUs. The main responsibility of top management is to generate the organisational conditions so that heads of the OUs (middle management) can carry out their work as effectively and efficiently as possible. In this regard, top management should support and promote decisive managerial practices such as performance assessment and staff motivation, to improve outcomes. Our study in fact shows that NICUs with a collaborative model have more robust performance measurement systems and more motivated staff than other models. Especially for performance evaluation, the data show great room for improvement in current practice (see MF2 in Table 1). The issue of performance assessment in intensive care unit is not new [55] but awareness of the need to increase the number of measures to be used, to include information on processes and structure in the evaluation system, and to compare the performance among different operating units is more recent [56, 57]. As for staff motivation, many studies find a relationship between staff motivation and outcome as well as between work environment and staff motivation [58, 59]. For top management, improving the work environment may be a promising strategy to improve patient safety and quality of care for at-risk newborns.

The second category of managers are heads of OUs (middle management), who are NICU team leaders and are in charge of NICU. In Italy, OU Heads have great power. They are responsible for running and organising the structure, managing clinical outcomes, managing human resources, and overseeing technical and financial targets. The coordination of health professionals in clinical and care processes, as well as the enhancement of employees, are key activities of middle managers in achieving quality performance. Creating teams, promoting coordination, and encouraging the involvement of collaborators (such as nurses) in decision-making processes are therefore necessary factors for a results-oriented leadership style. This style of leadership also increases employee motivation [60]. Previous empirical research has already shown that there is a positive relationship between the greater involvement of nurses in care and decision-making processes and neonatal outcomes [61, 62] and our results are consistent with these studies. The “collaborative” managerial model, which shows greater attention to the nursing role, displays better outcomes. However, even in this area (see MF3 and MF4 in Table 1), the results show room for improvement. Previous research that used PES-NWI in NICU shows in fact higher values in the areas we investigate [36, 63, 64]. Only the NICUs of Cluster 2 have values that are close to what was found in previous studies.

The third category of managers are regional health system managers. They are responsible for resource distribution and assessment, as well as overall policy such as determining the number of NICUs in area, the number of beds per NICU, etc. Our findings offer interesting indications for the development of policies capable of generating better performance in hospitals. First, effective performance assessment systems should be promoted. The systematic measurement of output and outcome, as well as the comparison of performance between hospitals and NICUs, allows public regional health system managers to identify best practices and to disseminate them throughout the territory. Indeed, policies of managerial change are required in order to restructure clinical and care processes based on the experiences of the best NICUs. Policies should also support the development of collaborative management methods and therefore help the management of hospitals in the most relevant managerial features (Performance measurement; Participation and support; Nursing foundations for quality of care; Motivation of medical and nursing staff). Of course, regional health system managers also need to help hospitals have access to appropriate tools for this purpose: policies to incentivise staff, criteria for selecting managerial roles, etc. Policies focused more on promoting effective managerial variables than on structural variables (beds or staff per bed) would certainly be innovative, at least in context of Italy. We found that NICU personnel endowment is not directly linked to managerial models. Of course, specific local elements such as staff allocation problems or lack of specialist skills among staff can impact on the allocation of personnel to NICU [20], but comparison with staff in other NICUs could be useful in drafting policy for reducing costs without compromising quality. Finally, the level of complexity of the cases is not a particular feature of any cluster. This implies that public policies can set up the NICU network consistently with local requirements, without needing to concentrate the most complex cases in particular OUs.

## Conclusion

NICU is one of the most expensive and complex areas in healthcare. Thus, health management needs to assess NICU strategically in order to develop models to improve outcomes. The wide variability of neonatal outcomes, determined by many factors, has in fact shifted focus towards the need to develop not only clinical practices but also effective managerial practices [13]. Our study provides insights useful for management in drafting the managerial characteristics of NICUs for the improvement of performance. To our knowledge, it is the first study to investigate the association between managerial models and neonatal outcomes. However, it is subject to certain limitations.

First of all, the study identifies five managerial features on the basis of the assessment systems developed by Øvretveit for WHO, but several other frameworks exist in literature. Furthermore, for each area of the Øvretveit’s model, different variables could be used. For example, we used the acuity score for measuring the complexity of cases, but some neonatology studies use the case mix index or the British Association of Perinatal Medicine standards [65]. There are also several ways of assessing performance, which can broadly be assessed by efficiency, patient satisfaction or quality of care [66]. However, in this study the focus is only on quality performance, which is considered the key dimension of hospital performance [22].

Finally, our research is performed in Italy. Future research is encouraged in other countries to confirm our findings.

## Supplementary information

**Additional file 1.** Questionnaire administered to medical staff, Questionnaire administered to medical staff who had been working in the NICU for at least 3 months in order to identified the system used to evaluate qualitative performance and the level of staff satisfaction.

**Additional file 2.** Questionnaire administered to nursing staff, Questionnaire administered to nursing staff who had been working in the NICU for at least 3 months in order to measure the working environment.

## Data Availability

The datasets analysed during the current study are available from the corresponding author on reasonable request.

## Abbreviations

NICU: Neonatal intensive care unit
WHO: World Health Organisation
SONAR: Observational Study in Neonatology: Assistance and CaRe
INN: Italian Neonatal Network
OU: Operational unit
MF: Managerial feature
PES-NWI: Practice Environment Scale of the Nursing Work Index
SD: Standard deviation
IVH: Severe Intraventricular Haemorrhage
PVL: Cystic periventricular leukomalacia
NEC: Necrotizing Enterocolitis
BPD: Pulmonary Bronchodysplasia
