# Supplementary material for: Managerial features and outcome in neonatal intensive care units: results from a cluster analysis
Source: BMC Health Serv Res. 2020 Oct 16;20:957. doi: 10.1186/s12913-020-05796-0 (PMC7565749; doi:10.1186/s12913-020-05796-0)
Supplement: Supplementary file 1 — Additional file 1. Questionnaire administered to medical staff, Questionnaire administered to medical staff who had been working in the NICU for at least 3 months in order to identified the system used to evaluate qualitative performance and the level of staff satisfaction. [file 12913_2020_5796_MOESM1_ESM.docx]

**Additional file 1**

**QUESTIONNAIRE ADMINISTERED TO MEDICAL STAFF**

**Please indicate the system used in your NICU to evaluate qualitative performance by choosing one of the five following statements:**

□ Quality measurement is sporadic

□ The individual evaluates his or her own performance using information made available by the hospital

□ Regular meetings between colleagues to discuss some clinical cases

□ We jointly evaluate all cases not following defined procedures

□ Periodical evaluation on quality indicators by external entities

**Please** **evaluate the level of satisfaction within your NICU:**

□ Very low

□ Low

□ Medium

□ High
