# Supplementary material for: Managerial features and outcome in neonatal intensive care units: results from a cluster analysis
Source: BMC Health Serv Res. 2020 Oct 16;20:957. doi: 10.1186/s12913-020-05796-0 (PMC7565749; doi:10.1186/s12913-020-05796-0)
Supplement: Supplementary file 2 — Additional file 2. Questionnaire administered to nursing staff, Questionnaire administered to nursing staff who had been working in the NICU for at least 3 months in order to measure the working environment. [file 12913_2020_5796_MOESM2_ESM.docx]

**Additional file 2**

**QUESTIONNAIRE ADMINISTERED TO NURSING STAFF**

The Practice Environment Scale of the Nursing Work Index (PES-NWI) - Lake ET. Development of the practice environment scale of the Nursing Work Index. Res Nurs Health. 2002;25(3):176–188.

**Please indicate your level of agreement for each statement (1 = strongly disagree; 2 disagree; 3 = agree; 4 = strongly agree)**

|  | **1** | **2** | **3** | **4** |
| --- | --- | --- | --- | --- |
| Staff nurses are involved in the internal governance of the hospital. |  |  |  |  |
| Opportunity for staff nurses to participate in policy decisions. |  |  |  |  |
| Many opportunities for advancement of nursing personnel. |  |  |  |  |
| An administration who listens to and responds to employee concerns. |  |  |  |  |
| A director of nursing highly visible and accessible to staff. |  |  |  |  |
| Career development/clinical ladder opportunity. |  |  |  |  |
| Nursing administrators consult with staff on daily problems and procedures. |  |  |  |  |
| Staff nurses have the opportunity to serve on hospital and nursing department committees. |  |  |  |  |
| A chief nursing executive equal in power and authority to other top level hospital executives. |  |  |  |  |
| Use of nursing diagnoses. |  |  |  |  |
| An active quality assurance program. |  |  |  |  |
| A preceptor program for newly hired RNs. |  |  |  |  |
| Nursing care is based on a nursing, rather than a medical, model. |  |  |  |  |
| Patient care assignments that foster continuity of care, i.e., the same nurse cares for the patient from one day to the next. |  |  |  |  |
| A clear philosophy of nursing that pervades the patient care environment. |  |  |  |  |
| Written, up-to-date nursing care plans for all patients. |  |  |  |  |
| High standards of nursing care are expected by the administration. |  |  |  |  |
| Active inservice/continuing education programs for nurses. |  |  |  |  |
| Working with nurses who are clinically competent. |  |  |  |  |
| A head nurse who is a good manager and leader. |  |  |  |  |
| A head nurse/supervisor who backs up the nursing staff in decision making, even if the conflict is with a physician. |  |  |  |  |
| Supervisors use mistakes as learning opportunities, not criticism. |  |  |  |  |
| A supervisory staff that is supportive of the nurses. |  |  |  |  |
| Praise and recognition for a job well done. |  |  |  |  |
| Enough staff to get the work done. |  |  |  |  |
| Enough registered nurses to provide quality patient care. |  |  |  |  |
| Adequate support services allow me to spend time with my patients. |  |  |  |  |
| Enough time and opportunity to discuss patient care problems with other nurses. |  |  |  |  |
| A lot of teamwork between nurses and doctors. |  |  |  |  |
| Physicians and nurses have good relationships. |  |  |  |  |
| Functional collaboration (joint practice) between nurses and physicians. |  |  |  |  |
